# Roles of oxalate-degrading bacteria in fungus-growing termite nests

**DOI:** 10.3897/BDJ.12.e130041

**Published:** 2024-08-19

**Authors:** Qibiao Sun, Jing Li, Shameer Syed, Xiaofang Li, Huatao Yuan, Bin Lian

**Affiliations:** 1 College of Life Sciences, College of Marine Science and Engineering, Nanjing Normal University, Nanjing, China College of Life Sciences, College of Marine Science and Engineering, Nanjing Normal University Nanjing China; 2 Jiangxi Province Key Laboratory of Watershed Ecological Process and Information, Jiujiang Key Laboratory of Fungal Resources Conservation and Utilization, College of Pharmacy and Life Sciences, Jiujiang University, Jiujiang, China Jiangxi Province Key Laboratory of Watershed Ecological Process and Information, Jiujiang Key Laboratory of Fungal Resources Conservation and Utilization, College of Pharmacy and Life Sciences, Jiujiang University Jiujiang China

**Keywords:** fungus-growing termite, *Termitomyces*, oxalotrophic bacteria, oxalate-carbonate pathway

## Abstract

Fungus-growing termite (FGT) nests possess an oxalate pool derived from termite input and fungal oxalogenesis. The effect of oxalate biotransformation in the termite nest on the symbiotic association between FGTs and *Termitomyces* fungi is poorly understood. Here, we measured the pH value, mineral composition, oxalate and carbonate contents, along with the abundance and composition of oxalotrophic bacteria (OxB) in termite nests. The results showed the community structures of OxB in different parts of the termite nest across fungus comb, termite nest wall and surface soil, were significantly different. The diversity of OxB in the fungus comb was significantly lower than that in the termite nest wall and surface soil. Results also showed the abundance of OxB in the fungus comb was higher than that in the termite nest wall and significantly lower than that in the surface soil. In addition, we isolated and screened an oxalotrophic bacterium *Methylobacterium* sp. TA1 from the fungus comb, which can degrade calcium oxalate and convert it into calcite. Our results from the perspective of oxalate biodegradation and transformation show that the oxalate-carbonate pathway driven by OxB in active termite nests can maintain stable microecological environments in termite nests and is beneficial to the symbiotic association between FGTs and *Termitomyces*.

## Introduction

Macrotermitinae (Isoptera, Termitidae), a subfamily of higher termites, is broadly distributed throughout East Asia, Southeast Asia and Africa (Eggleton 2010), also known as “fungus-growing termites (FGTs)” due to their ability in cultivating monocultures of a specific fungi (the species of the genus *Termitomyces*) (Anwar et al. 2020). FGTs have long been considered as ecosystem engineers, which are abundant and play an important role in soil nutrient dynamics and soil properties in the Tropics due to their activities (Jouquet et al. 2011, Khan and Ahmad 2018). FGTs are considered as a predominant decomposer of plant litter in dry savannahs, where they are responsible for around 20% of organic carbon degradation (Aanen and Eggleton 2005).

FGTs form a complex mutual relationship with the *Termitomyces* (Basidiomycota, Lyophyllaceae) that are obligatory for both partners (Aanen et al. 2009, Eggleton 2010). The symbiont *Termitomyces* fungi not only provide high nitrogen-containing food for FGTs, but also promote synergistic degradation and utilisation of lignin and cellulose; in return, FGTs provide favourable environment and nutrients for the growth and fruiting body formation of *Termitomyces* fungi (Taprab et al. 2005, Rouland-Lefèvre et al. 2006, Li et al. 2017). The FGT nests maintain a high humidity in the central nest, a constant temperature around 30℃, a high CO_2_ concentration and a low pH condition (Vesala et al. 2019, Yang et al. 2021), which may inhibit the growth of competitive fungi and nematodes that are harmful to the symbiotic system. In active fungus combs, the sponge-shaped structure piled up by termite faecal deposits, *Termitomyces* is the dominant fungi. Once the nest is abandoned by FGTs or the fungus comb incubated without active FGTs, some exotic fungi, especially species of *Xylaria* may arise and rapidly cover the fungus comb (Guedegbe et al. 2009, Visser et al. 2011a, Lee and Lee 2015). Therefore, the existence of FGTs is necessary for the survival of *Termitomyces* fungi in FGT nests (Moriya et al. 2005).

Although the symbiotic association between FGTs and *Termitomyces* started more than 30 million years (Nobre et al. 2011, Roberts et al. 2016), the maintenance mechanism of the symbiotic system has still not been fully unveiled. Studies showed that the microbes in fungus combs have a significant impact on the microenvironment of the termite nest benefitting the symbiotic association (Yin et al. 2019, Barcoto et al. 2020, Li et al. 2021, Yang et al. 2021). In addition, plant materials collected by termites usually contain large amounts of calcium oxalate (Franceschi and Nakata 2005). The transportation and feeding by termites on dead leaves and rhizomes of plants can lead to continuous enrichment of calcium oxalate in termite nests, as well as that derived from the metabolism of the growing *Termitomyces* fungi, making the termite nest an oxalate reservoir. The accumulation of polymorphous calcium oxalate has a negative impact on the diet and life activity of termites and their larvae, affecting the gastrointestinal digestion capacity and causing physical damage, etc. (Korth et al. 2006, Park et al. 2009). Consequently, this may seriously damage the symbiotic microenvironment of the termite nest. However, the metabolism of oxalate in the termite nest and its impact on the symbiotic association are unclear yet.

The bacteria utilising oxalate as the sole carbon and energy source in the soil are known as oxalotrophic bacteria (OxB), which degrade oxalate to formic acid and CO_2_, resulting in the formation of carbonate (the oxalate-carbonate pathway, OCP) in appropriate conditions (Braissant et al. 2004, Cailleau et al. 2005, Cailleau et al. 2011). As the main driver of OCP pathway, OxB are closely related to sustainable development and carbon capture and have attracted academic attention (Syed et al. 2020). However, the role of OCP derived by OxB in the patch of the termite nest has not been reported. Based on the abundant oxalate accumulated in termite nests, we speculated that the biodegradation of oxalate in termite nests may play an important role in the growth of *Termitomyces* fungi and activities of FGTs and in maintaining a healthy termite nest. The degradation of oxalate by OxB is mainly accomplished by two key enzymes, formyl-CoA transferase (FRC, encoded by the *frc* gene) and oxyl-CoA decarboxylase (OXC), when one oxalate molecular degradation consumes one hydrogen ion and produces one molecule of CO_2_ (Svedružić et al. 2005). The specific primer sets (frc171-F/frc306-R) for the amplification of the *frc* gene, designed by Khammar et al. (2009), allows us to perform quantitative research and functional analysis of OxB in termite nests.

In view of many possible environmental and biological factors involved in maintaining habitat balance of ant nests, this paper intends to analyse the related mechanism and scientific principles from the perspective of oxalate biodegradation and transformation. In this study, the oxalate and carbonate contents in different structures of active termite nests were determined by chemical analysis and the abundance and community structure of OxB in termite nests were analysed by quantitative PCR and high-throughput sequencing technologies. Additionally, the characteristics of oxalate degradation by OxB isolated and screened from the fungus comb were assessed. This study deepens our understanding of the community structure of OxB and their potential role in this special symbiotic system.

## Material and methods

### Site description and sampling

**Figure 1.**
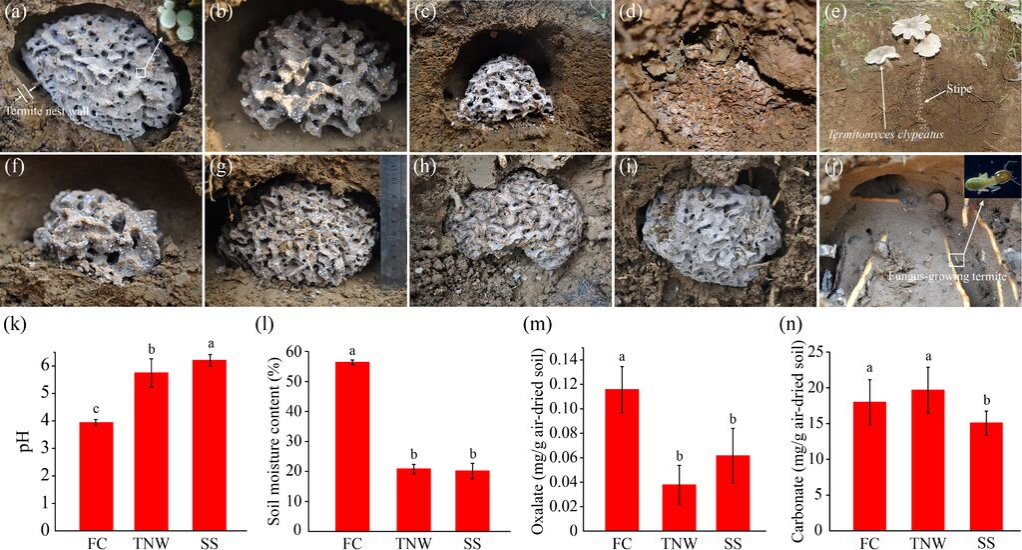
Different active fungus combs collected from the wild (a-i), sample pH (k) and moisture content (l) and the contents of oxalate (m) and carbonate (n) of different parts of the termite nest. *T.clypeatus* nodules (the enlarged region from the red rectangle in (a)) covered the surfaces of fungus combs. Fruiting bodies of *T.clypeatus* were linked with the subterranean fungus combs (e). The smooth solid termite nest walls and termites can be observed after removing the fungus comb (j). The letters on the error line represent significant differences, identified by Duncan’s test at p < 0.05. FC: fungal comb, TNW: termite nest wall, SS: surface soil.

The study site was located in Xianlin campus of Nanjing Normal University, Nanjing, Jiangsu Province, China, having subtropical monsoon climate with abundant rainfall (avg. annual precipitation of 1200 mm) and the annual average temperature of 15.4°C. The formation of fruiting bodies of *Termitomyces* fungi mainly occurs during July to September each year. The position of termite nests was estimated according to fruiting bodies of *Termitomyces* fungi. Termite nests were collected under *Broussonetiapapyrifera* (Linn.) L'Hér. ex Vent., *Cedrusdeodara* (Roxb.) G. Don, *Cinnamomumcamphora* (L.) Presl., *Cunninghamialanceolata* (Lamb.) Hook, *Pinusmassoniana* Lamb and *Trachycarpusfortunei* Wendl, respectively. Fungus combs were covered by hypha of *Termitomyces* fungi and distributed by lots of little white nodules (hyphal aggregates) in termite nests (Fig. 1a-i). The *Termitomyces* fungi cultured by the fungus-growing termite are *Termitomycesclypeatus* identified by amplifying and sequencing ITS region (ITS1-5.8S rRNA-ITS2) using the primer pair ITS1 (5'-TCCGTAGGTGAACCTGCGG-3') and ITS4 (5'-TCCTCCGCTTATTGATATGC-3') (Fig. 1e) and the FGTs found inside the termite nest are *Microtermespakistanicus*, identified by amplifying and sequencing mitochondrial cytochrome oxidase subunit II (COII) gene using the primer pair COII1 (5'-CAGATAAGTGCATTGGATTTCGG-3') and COII2 (5'-GTTTAAGAGACCAGTACTTG-3') (Fig. 1j). The soil (the surface of the termite nest, at a depth from 0 to 10 cm) above the termite nest was collected as the surface soil (SS). The soil (with a thickness of ~ 0.5 cm, Fig. 1a) inside of the termite nest was defined as the termite nest wall (TNW). The texture of TNW is very solid, differing from the surface soil significantly. A total of nine termite nests were sampled in this study. Samples from three different structures of the termite nest including the fungus comb (FC), TNW and SS were collected, respectively. Then, all samples were transported to the laboratory on ice. Each sample was homogenously mixed and divided into two subsamples, one subsample passed through a 2-mm nylon sieve was used for the determination of physical and chemical properties, the other was stored at –80°C for extracting soil genomic DNA.

### Physical and chemical properties assay

The moisture content of samples was calculated through loss of weight of 5 g sample oven-dried at 105°C for 12 h. Soil pH was determined at a 1:2.5 (w/v) soil-to-solution ratio using 1 M potassium chloride (KCl) solution. The oxalate content of samples was measured using a colourimetric method (Jin et al. 2007). Briefly, 25 ml of 1 M hydrochloric acid (HCl) were added to 10 g of dried sample for an overnight reaction on a reciprocating shaker and after shaking, pH was adjusted to 2 with 6 M HCl. The samples were centrifuged at 6000 rpm for 10 min and the supernatant was analysed using the method of Sun et al. (2019). The carbonate content was evaluated using a back-titration method according to the method of Sun et al. (2019). Mineral composition of samples was determined by X-ray powder diffraction (XRD) using an X-ray diﬀractometer BTX-526 (Olympus, Japan) using Co-Kα radiation (30 kV, 0.3 mA) from the 5° to 55° 2θ interval with 0.04° 2θ per step.

### OxB diversity in termite nests

The total genomic DNA of samples was extracted using PowerSoil^®^ DNA Isolation Kit (QIAGEN, Germany). DNA samples were transported to Shanghai Sangon Biotech in dry ice for Illumina MiSeq sequencing. Primer pair frc171-F (5'-CTSTAYTTCACSATGCTSAAC-3') and frc627-R (5'-TGCTGRTCRCGYAGYTTSAC-3') were used to specifically amplify *frc* fragments of OxB (Sun et al. 2019). PCR amplification was performed in triplicate using a 30 μl reaction mixture containing 15 μl 2× KAPA HiFi Hot Start Ready Mix (Takara, Japan), 1 μl of each primer (10 μM),= and 20 ng of template DNA. PCR was performed using the following programme: 1 cycle of denaturing at 95°C for 3 min, first 5 cycles of denaturing at 95°C for 30 s, annealing at 45°C for 30 s, elongation at 72°C for 30 s, then 20 cycles of denaturing at 95°C for 30 s, annealing at 55°C for 30 s, elongation at 72°C for 30 s and a final extension at 72°C for 5 min. The PCR products were checked using electrophoresis in 1% agarose gels. Library construction used universal Illumina adaptors and index sequences. Before sequencing, the DNA concentration of each PCR product was determined using a Qubit^®^ 2.0 Fluorometer (Invitrogen, USA) and it was quality controlled using an Agilent Bioanalyzer 2100 system (Agilent Technologies, USA). The amplicons from each reaction mixture were pooled in equimolar ratios, based on their concentration and sequenced on an Illumina MiSeq PE300 platform adopting a paired-end sequencing strategy.

Raw data were treated referring to Sun et al. (2019). In brief, raw reads were merged into complete sequences using FLASH 1.2.11 software (Magoč and Salzberg 2011). Barcode sequences were subsequently removed from each sample and sequences < 200 bp were discarded using Cutadapt v.1.14 software (http://cutadapt.readthedocs.io). The processed sequences were clustered into operational taxonomic units (OTUs) at 97% similarity using USEARCH (Edgar 2013). A representative sequence with the highest abundance in each OTU was selected for taxonomic assignment. Taxonomic annotation of representative sequences was performed using the BLASTn method in the National Center for Biotechnology Information (NCBI) non-redundant database and sequences with similarity < 90% and coverage < 90% were defined as unclassified. The raw data were uploaded to the NCBI SRA database (http://www.ncbi.nlm.nih.gov/sra) with the retrieval number SRR8835650.

### Isolation, identification and degradation characteristics of OxB

OxB on fungal combs were isolated by dilution coating on two-layer Schlegel solid media with calcium oxalate as the sole carbon source (Aragno 1992). Some larger colonies that produced transparent haloes were transferred to fresh Schlegel solid medium for further screened and purification. Finally, the isolate TA1 with the largest transparent halo was selected for subsequent studies. The micromorphology of the isolate TA1 was observed using a scanning electron microscope (SEM) S-3400 N (Hitachi, Japan). The isolate TA1 was also identified by 16S rRNA gene sequencing using the method of Sun et al. (2019). The 16S rRNA gene sequence of the isolate was deposited in GenBank with the accession number MK850375.

### Characteristics of biodegradation and biotransformation of oxalate by TA1

Since the isolate TA1 could not grow normally in liquid Schlegel medium, we selected modified King’s B medium to analyse the oxalate degrading ability of the isolate TA1 after trying different media. The components of modified King’s B medium (/l) were: CaC_2_O_4_ 50 g, tryptone 10 g, K_2_HPO_4_ 1.5 g, MgSO_4_·7H_2_O 1.5 g. The isolate TA1 was inoculated on solid Schlegel medium and incubated at 30°C for 5 days. Then, the bacteria were collected with sterile deionised water (SDW), washed thrice using SDW and diluted to 10^8^ cells per ml to serve as inoculum. The inoculum was inoculated into an Erlenmeyer flask (250 ml) with 100 ml modified King’s B medium (containing 25 mM CaC_2_O_4_) and incubated at 180 rpm at 30°C in an orbital shaker for 3 days. After incubation, the precipitates were collected and washed thrice with deionised water and anhydrous ethanol, respectively and dried at 60°C for mineralogical analysis. The SEM Zeiss-Supra55 (Zeiss, German) equipped with an energy dispersive X-ray energy spectrometer (EDS) was used to detect microscopic morphology and elemental composition of the precipitates and the X-ray diffractometer BTX-526 was used to detect mineral phases.

### Quantitative analysis of OxB and total bacteria

The primer pair frc 171-F and frc 306-R (5′-GDSAAGCCCATVCGRTC-3′) was used for quantifying *frc* copy number and primer pair 338F (5′-ACTCCTAGGGGGCGAGCAG-3′) and 518R (5′-ATTACCGCGGCGTGCTGG-3′) was used to detect the copy number of 16S rRNA gene in the total bacteria. The specific quantitative steps used were according to Sun et al. (2019).

### Statistical analysis

In R version 3.3.2, the *vegan* package was used to calculate α diversity indices (Chao1, Shannon, Simpson) and β diversity analysis (including the principal coordinate analysis (PCoA), Adonis analysis, canonical correspondence analysis (CCA) and Mantel test. The heatmap was generated using the *pheatmap* package. T-test and one-way ANOVA were performed using SPSS 20 (IBM, USA). Data are expressed as mean ± standard deviation.

## Results

### Physico-chemical properties of the termite nest

pH value, humidity, oxalate and carbonate contents in different structures of the termite nest were measured. The pH value of FC was 3.93 ± 0.01, significantly lower than that of TNW (5.74 ± 0.27) and SS (6.20 ± 0.22, Fig. 1k). The moisture of FC was 56.37% ± 0.80%, significantly higher than that of TNW (20.81% ± 1.55%) and SS (20.16% ± 2.59%, Fig. 1l). The oxalate content in the fungus comb was 0.12 ± 0.02 mg/g dry sample, which was significantly higher than that in the nest wall and surface soil (Fig. 1m). The highest calcium carbonate content was found in TNW (19.67 ± 3.22 mg/g soil), followed by FC (17.99 ± 3.14 mg/g soil) and SS (15.08 ± 1.65 mg/g soil, Fig. 1n).

**Figure 2.**
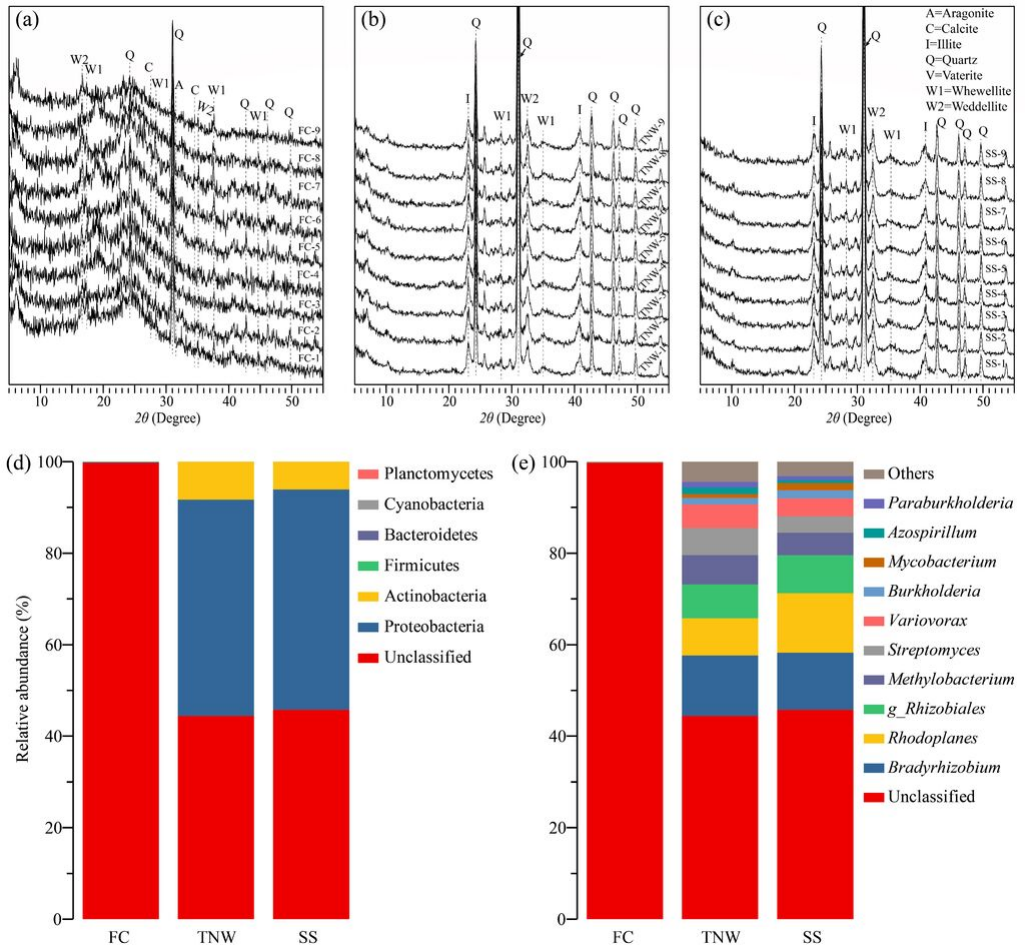
XRD patterns of soil mineralogical features of (a) FC, (b) TNW and (c) SS. Relative abundances of the phyla (a) and the top 10 genera (b) of OxB. Others, the remaining genera with lower relative abundance; Unclassified, no annotation information.

The results of XRD showed that the main mineral in different structures of termite nests was quartz, but oxalate minerals (whewellite and weddellite) were also detected (Fig. 2). The characteristic peaks of calcite and aragonite appeared in FC by XRD analysis, Fig. 2a), but not in TNW or SS, indicating crystallised calcium carbonate exist in termite nests. In addition, clay minerals, such as montmorillonite, were detected in FC and illite was detected in TNW and SS.

### Community structure of OxB in the termite nest

The diversity of OxB in FC, TNW and SS were determined using Illumina sequencing. After splicing and quality control of the raw data, a total of 2,512,669 valid sequences were obtained.The average sequence number obtained for each sample was > 29,000 with an average sequence length of 370 bp. The α-diversity indices of OxB in the termite nest are shown in Table 1.

The number of OTUs in FC was significantly lower than that in TNW and SS, which indicated that the species richness of OxB in FC was significantly lower than that in TNW and SS (Table 1). The Chao1 index showed that the predicted number of OTUs was greater than that of detected OTUs, indicating more OxB were unrevealed in the termite nest. The result of Shannon index also showed that the diversity of OxB in FC was significantly lower than that in TNW and SS.

**Figure 3.**
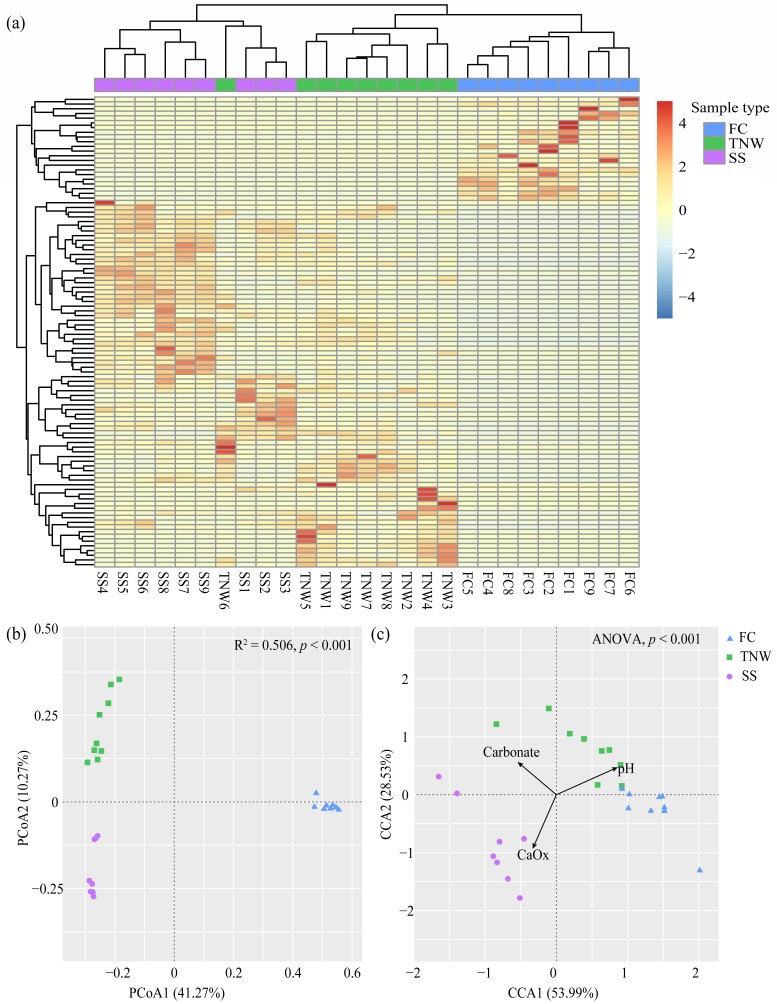
Heatmap of the top 100 OTUs (a), Principal coordinate ordination diagram (b) and Canonical constrained ordination diagram (c).

OTUs were assigned to six bacterial phyla, including Actinobacteria, Bacteroidetes, Cyanobacteria, Firmicutes, Planctomycetes and Proteobacteria. Amongst them, Cyanobacteria and Planctomycetes were found to contain *frc* for the first time. Cyanobacteria was only detected in SS. Firmicutes was only found in FC. Planctomycetes was found in SS and TNW. In SS and TNW, Proteobacteria and Actinobacteria were the dominant phyla, accounting for 41.80%–55.07% and 3.27%–19.97% of the total sequences, respectively. In FC, more than 99% of the sequences had no annotation information (Fig. 2d). A total of 61 species of OxB were annotated in the NCBI non-redundant database. Fig. 3e shows the composition and abundance of the top 10 dominant OxB in the termite nest, amongst which *Azospirillum*, *Bradyrhizobium*, *Burkholderia*, *Methylobacterium*, *Mycobacterium*, *Paraburkholderia*, *Rhodoplanes*, *Streptomyces* and *Variovorax* are the dominant genera.

We compared the differences of relative abundance of the first 100 dominant OTUs in different samples. The results showed that the composition of OxB in FC samples was significantly different from TNW and SS (Fig. 3a). The results of PCoA showed that the community structure of OxB amongst FC, TNW and SS was divided into three clusters with significant difference identified by ADONIS, based on Bray-Curtis distance (Fig. 3b). The results of CCA showed the environmental variables (pH, carbonate and oxalate) were associated with OxB communities (Fig. 3c), but only 18.63% of the total variance in OxB community structure was explained. The Mantel test, based on Pearson's product-motion correlation, further showed that pH value had a moderate positive correlation with OxB community (r = 0.390, *p* < 0.001), while oxalate and carbonate content had a weak positive correlation with OxB community (r = 0.105, *p* = 0.057; r = 0.124, *p* = 0.045).

**Figure 4.**
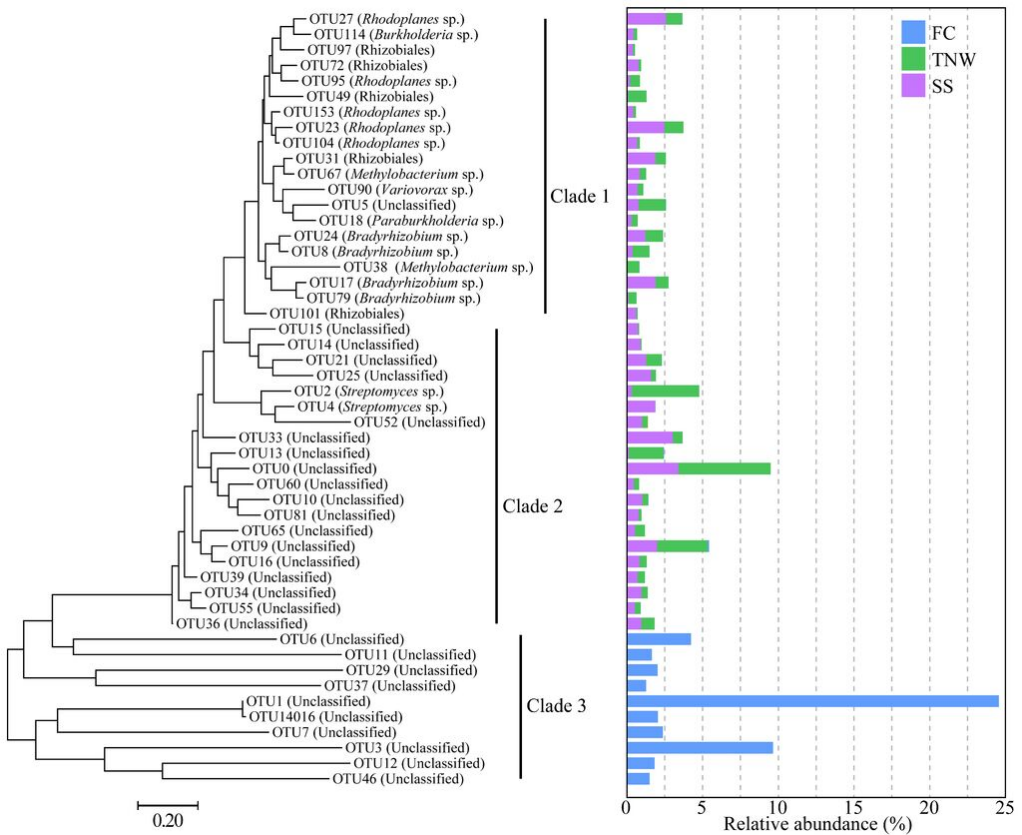
Phylogenetic relationships of the top 50 OTUs inferred using the Maximum Likelihood method (left) and their relative abundance in different samples (right). The evolutionary distances were computed using the Tamura-Nei model. All ambiguous positions were removed for each sequence pair. Evolutionary analyses were conducted in MEGA7.

In addition, phylogenetic relationships of the top 50 dominant OTUs and their relative abundance in different samples, based on *frc* sequence, were conducted (Fig. 4). The results showed that OTUs in FC differed from those in TNW and SS in *frc* sequences and relative abundance. OTU1, OTU3, OTU6, OTU7, OTU11, OTU12, OTU29, OTU37, OTU46 and OTU14016 also existed in FC and were also the dominant group, accounting for 50.72% of the total sequences.

### Population sizes of OxB and total bacteria in the termite nest

**Figure 5.**
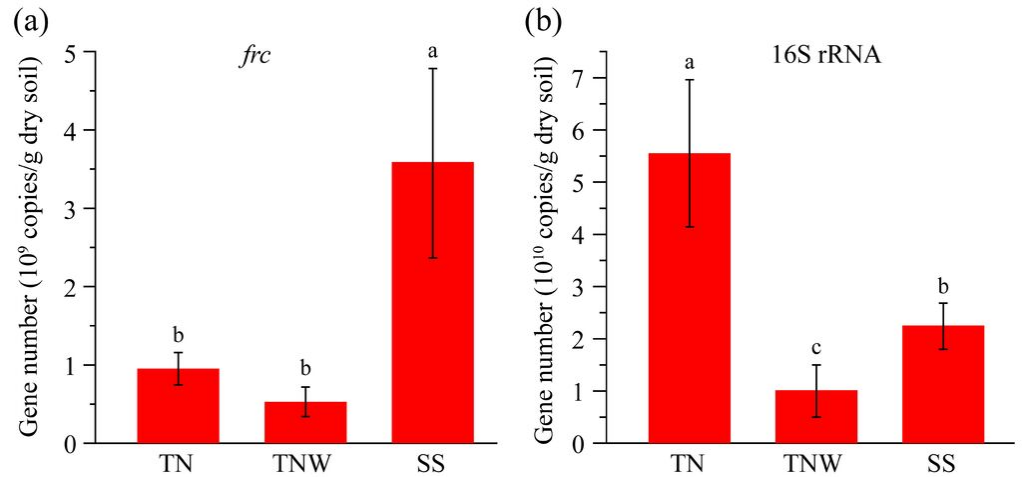
Gene copies of *frc* and 16S rRNA in soil samples determined by quantitative PCR. Values are means (± standard deviations) of nine individual samples. The letters on the error line represent significant differences, identified by Duncan’s post hoc test at *p* < 0.05.

The copy numbers of the total bacteria and OxB in FC, TNW and SS were determined by absolute quantitative PCR (Fig. 5). The number of OxB in FC was 9.87 × 10^8^ ± 2.03 × 10^8^ copies/g dry soil, higher than that in TNW (5.70 × 10^8^ ± 1.92 × 10^8^ copies/g dry soil), but significantly lower than that in SS (3.59 × 10^9^ ± 1.22 × 10^9^ copies/g dry soil). The number of bacteria in FC was (5.55 ± 1.41) ×10^10^ copies/g dry soil, significantly higher than that in TNW (1.01 × 10^10^ ± 0.50 × 10^10^ copies/g dry soil) and SS (2.24 × 10^10^ ± 0.44 × 10^10^ copies/g dry soil).

### Isolation and identification of OxB and determination of oxalate biotransformation

**Figure 6.**
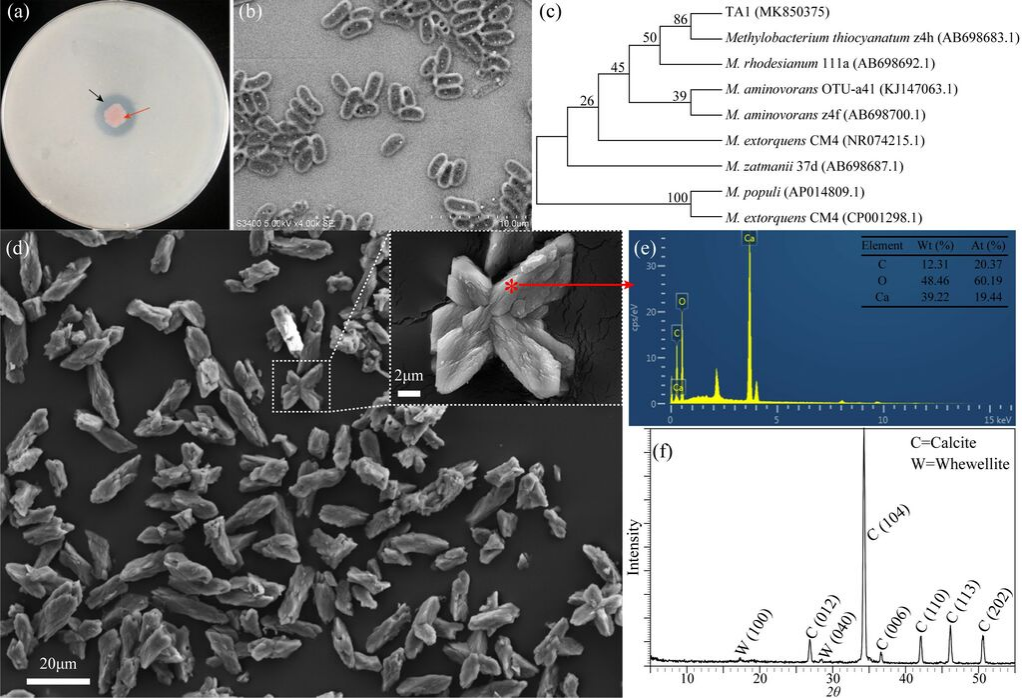
The colony growing on a medium with oxalate as sole source (a) and scanning electron micrograph of isolate TA1 (b). The phylogenetic tree, based on 16S rRNA gene sequence using the Neighbour-joining method, showed the phylogenetic relationship of the isolate TA1 was related with the species of *Methylobacteriumthiocyanatum* and bootstrap values calculated from 1000 repetitions are included at the nodes (c). SEM (d), EDS (e) and XRD (f) of crystals from calcium oxalate-containing liquid King’s B medium.

A short-rod-shaped oxalate degrading isolate TA1, identified as *Methylobacterium* sp., based on 16S rRNA gene sequencing, was isolated from the fungus comb (Fig. 6a-c). Liquid culture showed that the oxalate was completely degraded after incubation for 72 h (Suppl. material 1), a greater oxalate degradation capacity than *Streptomyces* sp. NJ10 from the report of Sun et al. (2019). The pH of the medium increased significantly during incubation. SEM imagery displayed that TA1 could form irregular rod-shaped or star-like secondary precipitates during the degradation of calcium oxalate (Fig. 6d). The EDS analysis showed that the precipitates were mainly composed of carbon, oxygen and calcium and its atomic number ratio was about 1:3:1 (Fig. 6e). Further analysis by XRD showed the precipitates were calcite (Fig. 6f).

## Discussion

FGT-*Termitomyces* fungal-bacterial interactions are the key to maintain the normal functioning of the termite nest (Aanen et al. 2009, Sawhasan et al. 2012, Barcoto et al. 2020, Li et al. 2021, Yang et al. 2021). In this study, we showed the diversity of OxB in FGT nests. It presents a scenario that the composition and community structure of OxB in the fungus comb was significantly different from the termite nest wall and surface soil. It suggests the termite nest, especially the fungus comb, was an important reservoir of OxB. Although the species number of OxB in the fungus comb was low, the population size of OxB in the fungus comb was higher than that in the termite nest wall (Fig. 5). Moreover, the community structures of OxB in different parts of the termite nest were different (Figs 2, 3). For example, the relative abundance of the top 10 OTUs in the fungus comb occupied more than half of the proportion. The termite nest perhaps only selects and holds specific microbial species to form efficient cooperation. OxB in the fungus comb may be carried in when termites are out foraging or from intestinal excreta. The activities of FGTs can affect the structure and composition of OxB in the termite nest, while *Termitomyces* fungi may also affect the community structure of OxB through symbiosis with termites.

Although roles of many bacteria in the termite nest has been documented (Visser et al. 2011b, Sawhasan et al. 2012, Beemelmanns et al. 2017, Yin et al. 2019, Li et al. 2021), we first reported the diversity of OxB in the termite nest and their potential impact on the FGT-*Termitomyces* fungal symbiotic association. The degradation of OxB can mitigate the impact of oxalate accumulation on feeding of FGTs (especially older workers) in termite nests and regulate the microenvironment due to alkalisation and CO_2_ release in the process of oxalate degradation by OxB (Svedružić et al. 2005). The Oxalotrophic *Streptomyces*, confirmed by Sahin (2004) and Sun et al. (2019), detected in this study may be a generalist. For example, the study of Li et al. (2021) showed *Streptomyces* strains have an inhibitory effect on entomopathogen (*Metarhiziumanisopliae*) and competitive fungi (*Xylaria* spp.).

In this study, we found that the oxalate content in the fungus comb was significantly higher than that in the termite nest wall and surface soil (Fig. 2m), which was associated with the foraging behaviour of termites and extracellular secretion of *Termitomyces* fungi that promoted the accumulation of oxalate. Oxalate catabolism by OxB is a process of hydrogen ion consummation and leads to alkalisation of the medium *in vitro* (Suppl. material 1), also documented in previous studies (Braissant et al. 2002, Sahin 2004, Svedružić et al. 2005, Sun et al. 2019). The pH value of termite nests is from 4.0 to 5.0 (Yang et al. 2021), which is the optimal pH environment for the growth of *Termitomyces* fungi and inhibits the overgrowth of competitive fungi. Therefore, the degradation of oxalate by OxB may play an important role in maintaining a dynamic equilibrium of physical and chemical conditions.

Some OxB produce carbonate precipitates in the process of oxalate degradation (Cailleau et al. 2005). Our result also showed that the degradation, in vitro, of oxalate in media can form carbonate precipitates. The *Methylobacterium* sp. TA1 isolated from the fungus comb can induce the formation of carbonate precipitate (calcite) in the process of calcium oxalate degradation (Fig. 6). The carbonate content in the termite nest is an important factor indirectly reflecting the metabolic intensity and transformation characteristics of oxalate. The presence of carbonates (including calcite and aragonite) in the fungus comb suggests that the degradation of oxalate by OxB may contribute to this. Carbonate minerals existing in the termite nest have been documented, but the original of carbonates is still a controversial issue (Liu et al. 2006, Mujinya et al. 2011, Van Thuyne et al. 2021). This study also found that the termite nest wall was hard and glazed and contained high carbonate content, but carbonate minerals were not detected by XRD (Figs 1, 2), perhaps most of the carbonate in the wall of the nest being amorphous. Calcareous materials are usually used to build nests by termites as a cementing agent (Mujinya et al. 2011), which indicates that the final product of OCP, calcium carbonate, is likely to participate in the construction of the internal structure of the termite nest as a biological building material. Amorphous carbonate may improve the cohesion and strength of the termite nest wall, making it less likely to collapse, making the structure of the nest safer and more stable (Liu et al. 2006) and becoming a potential carbon sequestration patch.

The carbonate produced through OCP in the fungus comb may also play an important role in buffering and maintaining the pH value of the termite nest environment. *Xylarianigripes* is the most important competitive fungus in active termite nests, most of its carbon sources overlapping with those of *Termitomyces* fungi (Visser et al. 2011a). In this study, we observed fruiting bodies of *X.nigripes* formed on fungus combs collected from the field and stored in the refrigerator at 4°C (Suppl. material 2). Active termite nests are usually maintained at a stable temperature (ca. 30°C) and low pH (Vesala et al. 2019, Yang et al. 2021), which benefits the growth of *Termitomyces* fungi. At the same time, the termite nest usually keeps a high concentration of CO_2_ (around 2%), which can inhibit the growth of xylariaceous fungi. In addition to the special structure of the termite nest, the respiration of termites and microorganisms, CO_2_ released by oxalate degradation may play important roles in regulating the temperature and pH in the termite nest. We isolated *T.clypeatus* from fresh fungus combs and *X.nigripes* from the fungus comb stored in the laboratory. The results showed that the growth of *X.nigripes* was affected by low pH and high concentration of CO_2_ (Suppl. materials 3, 4).

**Figure 7.**
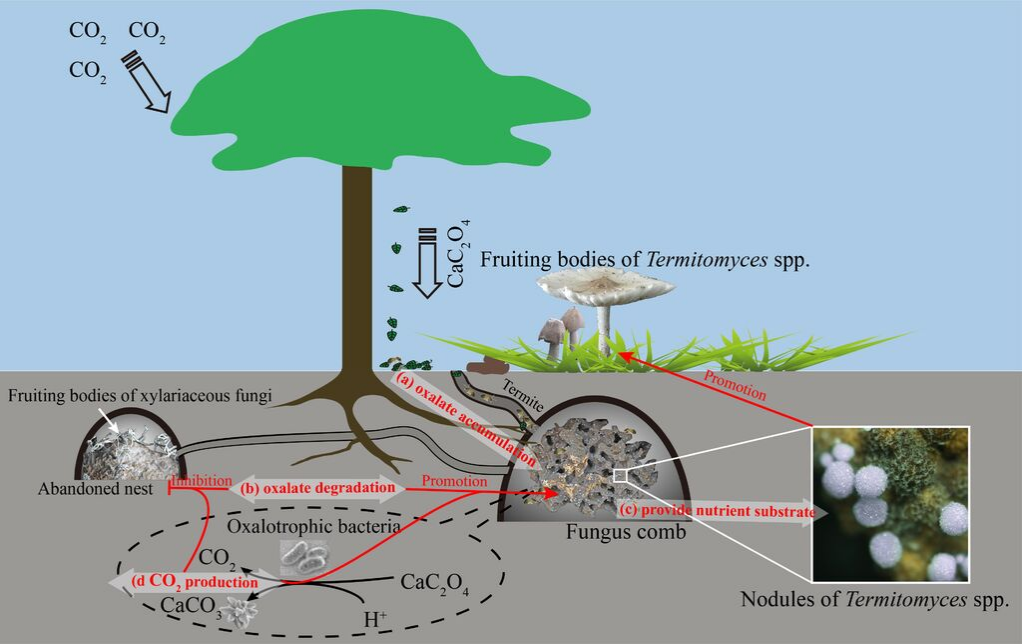
Schematic diagram of microecological relationships in termite nests. a) Fungus-growing termites carry calcium oxalate into their nests and accumulate oxalotrophic bacteria (OxB); b)The degradation of oxalate by OxB can reduce the toxicity of oxalate, regulate the pH of the fungus comb, form carbonates and thus maintain conditions (pH, temperature, humidity, CO_2_ concentration etc.) conducive to the growth of *Termitomyces* fungi; c) The termite nest provides a rich nutrient substrate for the growth of *Termitomyces* fungi, while the fungi provide mycelium rich in nutrition (especially nitrogen source) for termites; d) CO_2_ by the metabolism of termites, *Termitomyces* fungi and OxB in the active termite nests are the inhibitory factors for the growth of xylariaceous fungi in the active termite nest to some extent.

In summary, there is an abundance of oxalate and OxB in the active termite nest. The biodegradation of oxalate plays a role in the symbiotic association between FGTs and *Termitomyces* fungi. Ca^2+^ and CO_2_ released by the biodegradation of oxalate can promote the formation of carbonates, which important for the regulation of active termite nest microenvironment. In addition, the biodegradation of oxalate is an important, but underrated inorganic carbon sequestration potential process in termite nests as well (Fig. 7). From the perspective of oxalate biodegradation and transformation, this study proved that the biotransformation by OxB in the termite nest plays a role in the maintenance of FGTs and *Termitomyces* symbiotic system. The carbon sink process driven by OCP in the termite nest deserves further investigation.

## Conclusions

Oxalate and OxB are present in large amounts in the active termite nest. The biodegradation of oxalate plays a role in the symbiotic association between FGTs and *Termitomyces*. Ca^2+^ and CO_2_ released by the biodegradation of oxalate promote the formation of carbonates, which important for the regulation of active termite nest microenvironment. From the perspective of oxalate biodegradation and transformation, this study proved that the biotransformation by OxB in the termite nest plays a role in the maintenance of FGT-*Termitomyces* symbiotic system. The carbon sink process driven by OCP in the termite nest deserves further investigation.

## Supplementary Material

8D8E7EBB-96D1-543F-9558-59C01BF5158F 10.3897/BDJ.12.e130041.suppl1 Supplementary material 1 Fig. S1 Data type images Brief description Characteristics of calcium oxalate biodegradation by the isolate TA1 incubated in the modified King’s B liquid medium at 30°C, 180 rpm for 72 h. File: oo_1067289.tif https://binary.pensoft.net/file/1067289 QS, JL, SS, XL, HY and BL

F08B00B5-23A7-5018-A835-1040B8934887 10.3897/BDJ.12.e130041.suppl2 Supplementary material 2 Fig. S2 Data type images Brief description *X.nigripes* growing on fungus combs that were stored in the refrigerator at 4°C for 45 days. File: oo_1067292.tif https://binary.pensoft.net/file/1067292 QS, JL, SS, XL, HY and BL

F7C1D942-55EF-5035-A537-C4438F8A1DEA 10.3897/BDJ.12.e130041.suppl3 Supplementary material 3 Fig. S Data type images Brief description Growth characteristics of *T.clypeatus* and *X.nigripes* at different pH conditions at 25°C for 14 days. File: oo_1067295.tif https://binary.pensoft.net/file/1067295 QS, JL, SS, XL, HY and BL

5B9473F1-E5EB-52A5-9FA5-C57702BD3221 10.3897/BDJ.12.e130041.suppl4 Supplementary material 4 Fig. S4 Data type images Brief description Growth characteristics of *T.clypeatus* and *X.nigripes* at different CO_2_ concentrations at 25°C for 14 days. File: oo_1067297.tif https://binary.pensoft.net/file/1067297 QS, JL, SS, XL, HY and BL

## Figures and Tables

**Table 1.** The α-diversity indices of different groups (n = 9)

Samples	α-diversity indices			
	OTU number	Simpson	Shannon	Chao1
FC TNW SS	1334 ± 351b 2812 ± 415a 3064 ± 439a	0.10 ± 0.03a 0.02 ± 0.01b 0.01 ± 0.00b	4.10 ± 0.45b 5.89 ± 0.16a 6.00 ± 0.13a	1900 ± 506b 3576 ± 558a 3913 ± 488a

The letters in each column represent significant differences, identified by Duncan’s post hoc test at *p* < 0.05.
