# Supplementary figures and images for: Roles of oxalate-degrading bacteria in fungus-growing termite nests
Source: Biodivers Data J. 2024 Aug 19;12:e130041. doi: 10.3897/BDJ.12.e130041 (PMC11347878; doi:10.3897/BDJ.12.e130041)

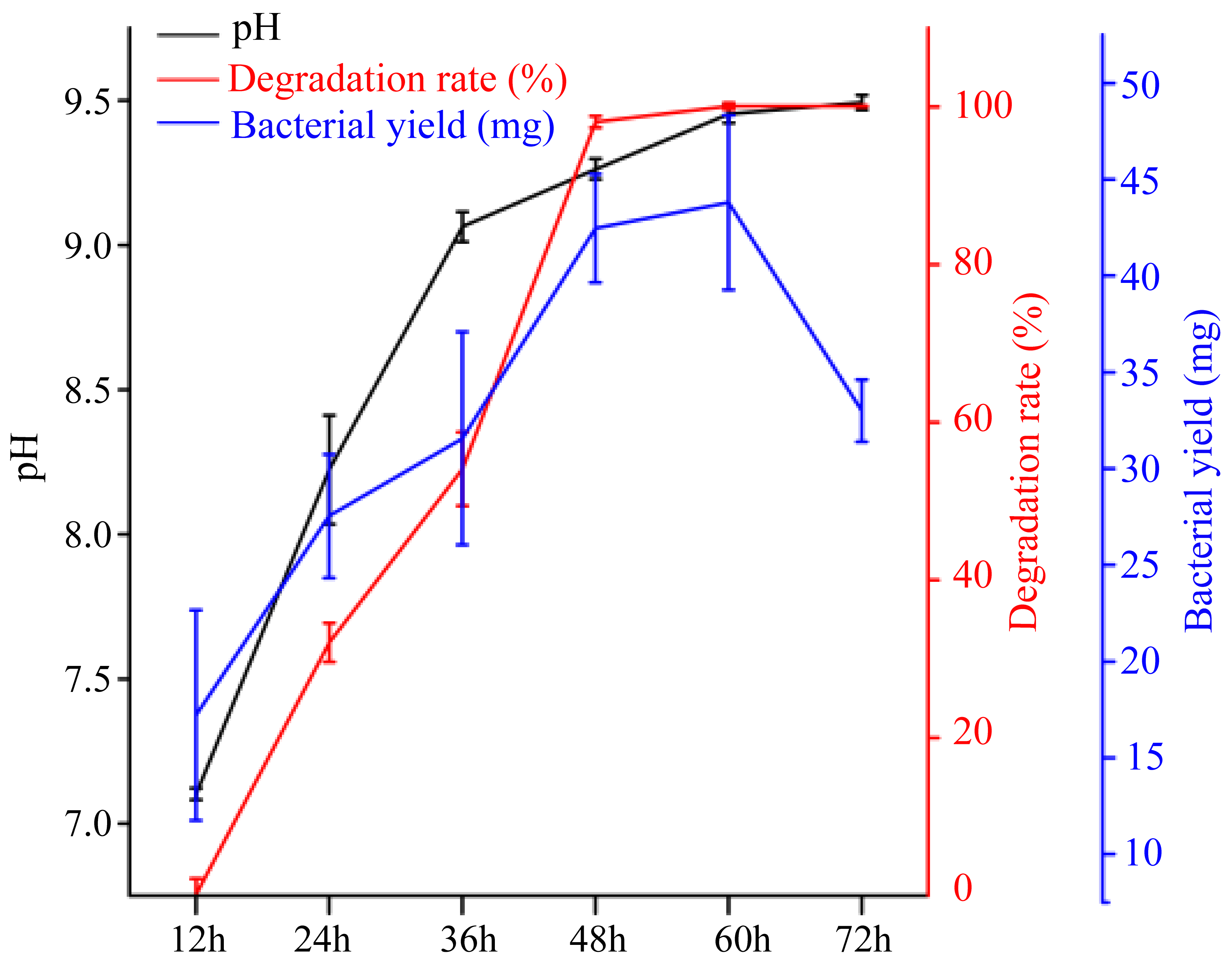

Supplement: Supplementary material 1 — Fig. S1 [file bdj-12-e130041-s001.tif]

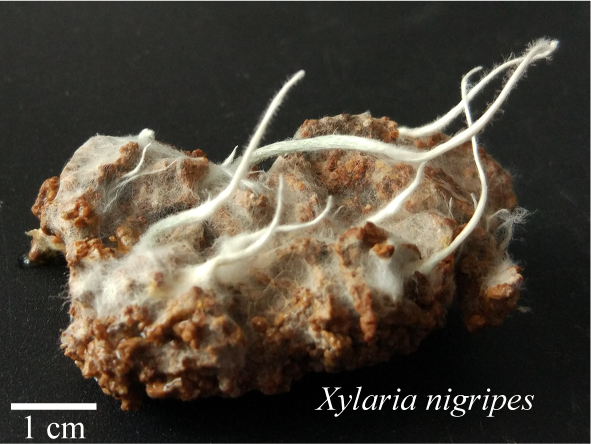

Supplement: Supplementary material 2 — Fig. S2 [file bdj-12-e130041-s002.tif]

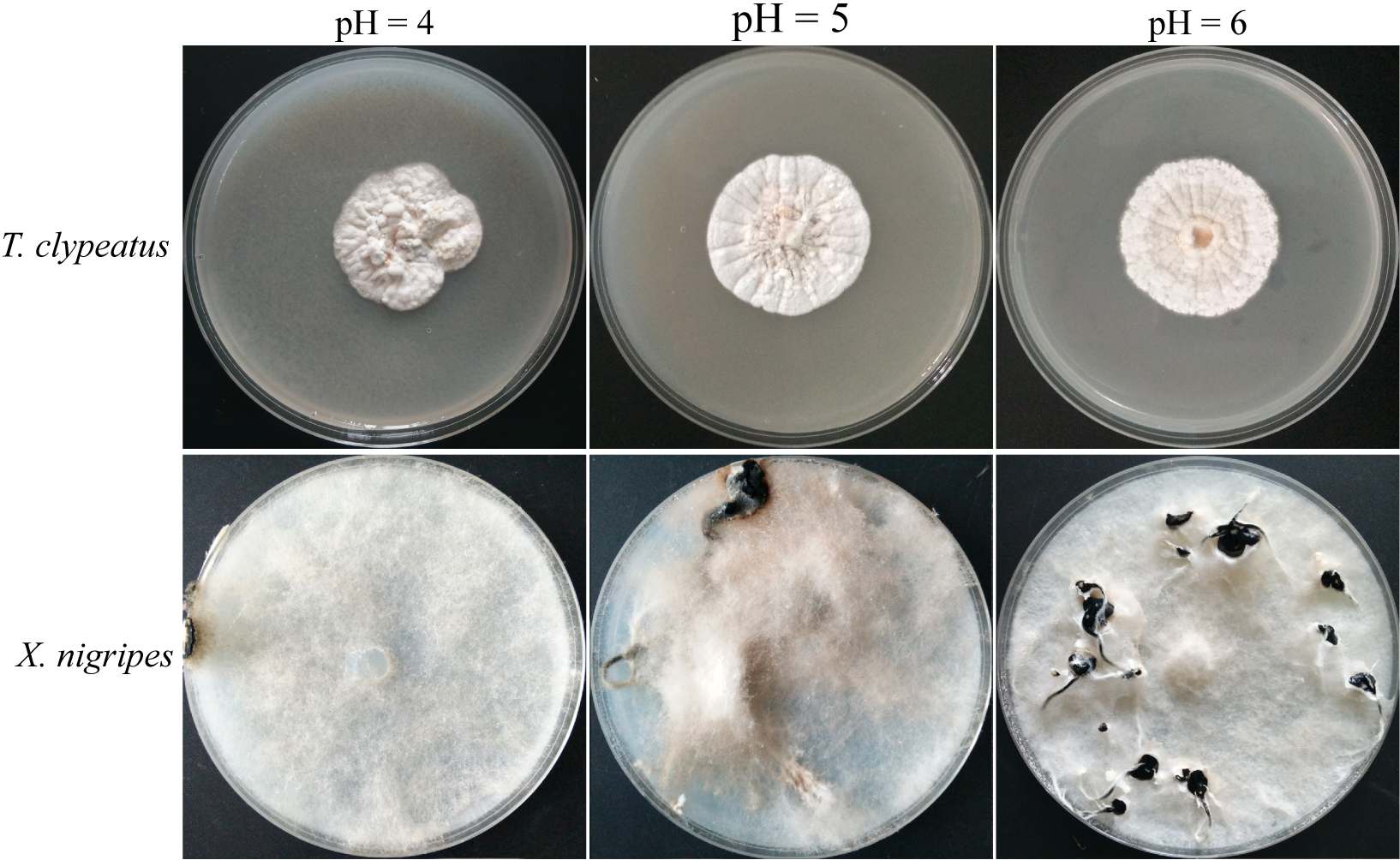

Supplement: Supplementary material 3 — Fig. S [file bdj-12-e130041-s003.tif]

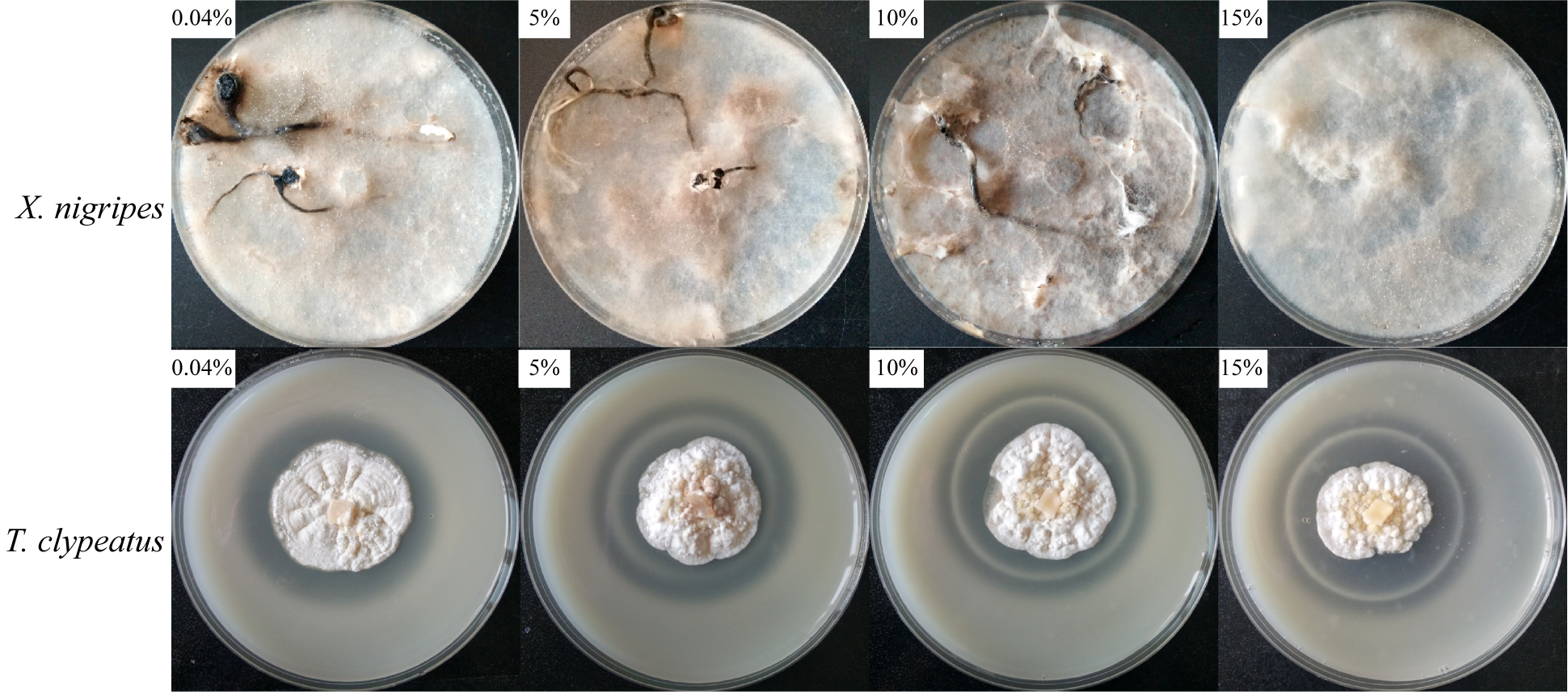

Supplement: Supplementary material 4 — Fig. S4 [file bdj-12-e130041-s004.tif]
